# Iron Oxide Nanoparticles as Autophagy Intervention Agents Suppress Hepatoma Growth by Enhancing Tumoricidal Autophagy

**DOI:** 10.1002/advs.201903323

**Published:** 2020-06-09

**Authors:** Yuexia Xie, Jiana Jiang, Qianyun Tang, Hanbing Zou, Xue Zhao, Hongmei Liu, Ding Ma, Chenlei Cai, Yan Zhou, Xiaojing Chen, Jun Pu, Peifeng Liu

**Affiliations:** ^1^ State Key Laboratory of Oncogenes and Related Genes Shanghai Cancer Institute Ren Ji Hospital School of Medicine Shanghai Jiao Tong University Shanghai 200032 China; ^2^ Central Laboratory Ren Ji Hospital School of Medicine Shanghai Jiao Tong University Shanghai 200127 China; ^3^ Micro–Nano Research and Diagnosis Center Ren Ji Hospital School of Medicine Shanghai Jiao Tong University Shanghai 200127 China

**Keywords:** hepatoma therapy, iron oxide nanoparticles, iron transport systems, tumoricidal autophagy

## Abstract

The combined treatment with nanoparticles and autophagy inhibitors, such as chloroquine (CQ) and hydroxychloroquine (HCQ), is extensively explored for cancer therapy. However, the toxicity of autophagy inhibitors and their unselective for tumoricidal autophagy have seriously hindered the application of the combined treatment. In this study, a carboxy‐functional iron oxide nanoparticle (Fe_2_O_3_@DMSA) is designed and identified to significantly exert an antitumor effect without adding CQ or HCQ. Further investigation indicates that the effective inhibition effect of Fe_2_O_3_@DMSA alone on hepatoma growth is triggered by inhibiting the fusion of autophagosomes and lysosomes to enhance tumoricidal autophagy, which is induced by intracellular iron‐retention‐induced sustained reactive oxygen species (ROS) production. Furthermore, in two hepatoma‐bearing mouse models, Fe_2_O_3_@DMSA alone effectively suppresses the growth of tumors without obvious toxic side effects. These studies offer a promising strategy for cancer therapy.

## Graphical abstract

Carboxy‐functional iron oxide nanoparticles (Fe_2_O_3_@DMSA) trigger intracellular iron‐retention‐induced sustained reactive oxygen species (ROS) production to activate autophagy but block the fusion of autophagosomes and lysosomes, which efficiently inhibits hepatoma growth through enhancing tumoricidal autophagy in vitro and in vivo. Fe_2_O_3_@DMSA shows potential clinical application for hepatoma therapy.

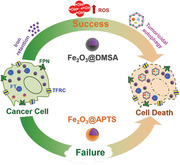

Among primary liver cancers, hepatocellular carcinoma (HCC) accounts for 75–85% of the diagnosed cases. Although great progress has been made in the treatment of HCC, patients with advanced HCC are not eligible for radical treatment such as hepatectomy or liver transplantation.^[^ ^1^ ^]^ Until recently, sorafenib, the only drug approved by Food and Drug Administration (FDA) for such patients, also only provided an average of 3 months of survival benefits.^[^ ^1^ ^]^ Moreover, these patients have always suffered from the low efficiency, high recurrence, high systemic toxicity, and severe effects on quality of life.^[^ ^2^ ^]^ Therefore, it is imperative to develop an effective drug for the therapy of HCC.

Recently, we found that iron oxide nanoparticles (IONPs), widely used as drug delivery carrier and contrast agents,^[^ ^3^ ^]^ possessed an intrinsic and directed cytostatic efficiency for HCC. During previous studies, IONPs realized antitumor efficiency based on the enhancement of Fenton‐like reactions and reactive oxygen species (ROS) generation.^[^ ^4^, ^5^, ^6^ ^]^ However, in our research, it has been confirmed that IONPs could be applied as autophagy intervention agents to directly suppress hepatoma by interfering with autophagy flux and inducing tumoricidal autophagy.

Despite various nanoparticles, including IONPs, copper–palladium alloy nanoparticles, calcium‐phosphate‐based nanoparticles, lipid‐coated Poly(lactic‐co‐glycolicacid) (PLGA) nanoparticles, and gold nanoparticles, have been applied to treat tumors by interfering with the autophagy process. It should be emphasized that their therapeutic efficiency can only be realized through the combined treatment with nanoparticles and autophagy inhibitors, such as chloroquine (CQ) and hydroxychloroquine (HCQ).^[^ ^7^, ^8^, ^9^, ^10^, ^11^ ^]^ Moreover, it must be noted that both CQ and HCQ can cause a serious retinopathy; therefore, the combined antitumor therapies with nanomaterial plus autophagy inhibitor (CQ or HCQ) have been greatly limited in the clinical application.^[^ ^12^, ^13^ ^]^ Herein, we discovered that Fe_2_O_3_@DMSA (carboxyl functional IONPs) exhibited a remarkable antitumor efficiency through directly interfering with the autophagy process without adding any autophagy inhibitors including CQ and HCQ. Subsequently, we further illustrated that Fe_2_O_3_@DMSA significantly promoted iron‐deposition‐induced sustained ROS accumulation, which triggered tumoricidal autophagy and effectively inhibited the growth of hepatoma (**Scheme** **1**). These results verified that Fe_2_O_3_@DMSA alone could be applied as an autophagy intervention agent to efficiently suppress hepatoma by directly enhancing tumoricidal autophagy.

**Scheme 1:**
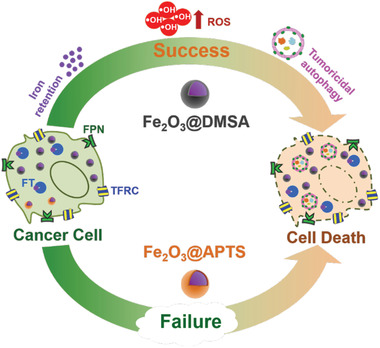
The carboxy‐functional iron oxide nanoparticles (Fe_2_O_3_@DMSA) significantly impacts on the iron transport system (TFRC, FT, and FPN) and promotes the retention of intracellular iron, resulting in excessive ROS‐induced tumoricidal autophagy.

We first employed Fe_2_O_3_@DMSA (carboxy‐functional IONPs) and Fe_2_O_3_@APTS (amine‐functional IONPs) with similar size and morphology to comparatively research their interaction with hepatoma cells. The morphologies of Fe_2_O_3_@DMSA and Fe_2_O_3_@APTS were characterized by transmission electron microscopy (TEM) (**Figure** **1**A,B) and scanning electron microscopy (SEM) (Figure 1C,D). We found that they exhibited similar shape and size with ≈10 nm. Atomic force microscope (AFM) analysis further showed that the sizes of Fe_2_O_3_@DMSA and Fe_2_O_3_@APTS were about 10 nm (Figure S1, Supporting Information). Fourier transform infrared (FTIR) spectroscopy was used to confirm that the meso‐2,3‐dimercaptosuccinic acid (DMSA) and 3‐aminopropyltriethoxysilane (APTS) were successfully coated on the Fe_2_O_3_. The FTIR spectra of Fe_2_O_3_@DMSA and Fe_2_O_3_@APTS were shown in Figure 1E. For Fe_2_O_3_@DMSA, the band at 1584 cm^−1^ corresponded to the COO^−^ stretching vibration, which revealed the existence of DMSA. The peaks at 631 and 583 cm^−1^ were assigned to Fe—O stretching vibration. For Fe_2_O_3_@APTS, the peak at 1626 cm^−1^ was attributed to bending vibration of —NH_2_. The peaks at 1097 and 1018 cm^−1^ were due to the Si—O bond, which confirmed the success of the silanization process. The peaks at 631 and 581 cm^−1^ were due to the Fe—O bond. Therefore, FTIR results confirmed the successful surface modification of Fe_2_O_3_ nanoparticles with DMSA and APTS, respectively. The zeta potentials of Fe_2_O_3_@DMSA and Fe_2_O_3_@APTS were about −40.3 ± 5.42 and 30.8 ± 4.38 mV, respectively (Figure 1F).

**Figure 1:**
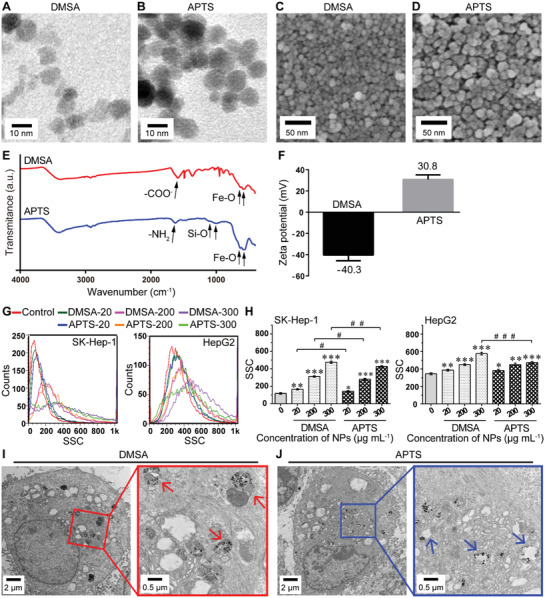
Characterizations of Fe_2_O_3_@DMSA and Fe_2_O_3_@APTS, and their internalized distribution. A,B) TEM images of Fe_2_O_3_@DMSA and Fe_2_O_3_@APTS. C,D) SEM images of Fe_2_O_3_@DMSA and Fe_2_O_3_@APTS. E) FTIR spectra of Fe_2_O_3_@DMSA and Fe_2_O_3_@APTS. F) Zeta potential of Fe_2_O_3_@DMSA and Fe_2_O_3_@APTS. G,H) Cellular uptake of Fe_2_O_3_@DMSA and Fe_2_O_3_@APTS in SK‐Hep‐1 and HepG2 cells were determined by flow cytometry after exposure for 24 h at the doses of 20, 200, 300 µg mL^−1^, respectively. The data represented mean ± standard deviation (SD). *p < 0.05, **p < 0.01, and ***p < 0.001 compared with control. ^#^ *p* < 0.05, ^##^ *p* < 0.01, and ^###^ *p* < 0.001 between the indicated groups. I) TEM images of internalized Fe_2_O_3_@DMSA. The red arrows indicated early autophagic vesicles with double membrane. J) TEM images of internalized Fe_2_O_3_@APTS. The blue arrows indicated degradative autophagic vesicles with single membrane. DMSA denotes Fe_2_O_3_@DMSA. APTS denotes Fe_2_O_3_@APTS.

To research the interaction of Fe_2_O_3_@DMSA and Fe_2_O_3_@APTS with hepatoma cells, we first detected the cellular uptake with flow cytometry in SK‐Hep‐1 and HepG2 cells. As shown in Figure 1G,H, the side‐scattered intensity (reflecting cellular uptake) increased in a dose‐dependent manner. However, the intracellular accumulation of Fe_2_O_3_@DMSA was significantly higher than that of Fe_2_O_3_@APTS, implying that Fe_2_O_3_@DMSA could enhance cellular uptake efficiency. Subsequently, we further researched their uptake path by TEM and confirmed that Fe_2_O_3_@DMSA and Fe_2_O_3_@APTS were internalized in the cellular cytoplasm and trapped in vesicles, suggesting that these two IONPs were internalized via endocytosis (Figure 1I,J).

Based on the higher cellular uptake efficiency of Fe_2_O_3_@DMSA, we next hypothesized that hepatoma cells treated with Fe_2_O_3_@DMSA would retain more soluble iron than those treated with Fe_2_O_3_@APTS, and thus exhibit a higher vulnerability to ROS with increased cell death. We observed a modest but significant increase in the intracellular iron content for Fe_2_O_3_@DMSA‐treated cells (Fold change (FC) = 1.170 ± 0.040, *p* = 0.0022), but no significant difference in Fe_2_O_3_@APTS‐treated cells (FC = 1.057 ± 0.037, *p* = 0.0678) at early incubation stages (8 h). With the incubation time prolonging to 24 h, although both treated groups exhibited higher intracellular iron levels compared with untreated group, Fe_2_O_3_@DMSA‐treated cells exhibited a dramatically higher iron content than Fe_2_O_3_@APTS‐treated cells (FC = 1.468 ± 0.031 vs 1.128 ± 0.017, *p* < 0.0001) (**Figure** **2**A). Subsequently, we further evaluated gene‐expression‐level‐related iron management. As expected, the greater increases in intracellular iron levels in Fe_2_O_3_@DMSA‐treated cells corresponded to the higher iron internalization transferrin receptor (TFRC) and storage (ferritin heavy chain 1 (FTH1) and ferritin light chain (FTL)) but lower iron export ferroportin (FPN) than that in Fe_2_O_3_@APTS‐treated cells, indicating that iron transport systems contributed to intracellular iron retention in Fe_2_O_3_@DMSA treatment (Figure 2B–E). Furthermore, cellular proliferation assay further showed that Fe_2_O_3_@APTS itself with rarely iron retention in cells exhibited a little cytotoxicity even at high concentration (400 µg mL^−1^) with an half maximal inhibitory concentration (IC_50_) of ≈2201.0 µg mL^−1^, while Fe_2_O_3_@DMSA exhibited much higher cytotoxicity with an IC_50_ of ≈494.2 µg mL^−1^ (Figure 2F). Lactate dehydragenase (LDH) leakage assay also demonstrated that Fe_2_O_3_@DMSA displayed higher cytotoxicity on hepatoma cells than Fe_2_O_3_@APTS (Figure S2, Supporting Information).

**Figure 2:**
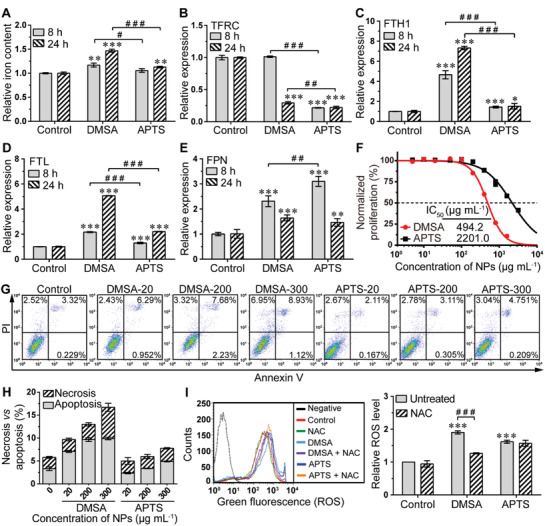
Intracellular soluble iron retention resulted in excess ROS and enhanced a higher cellular vulnerability with increased cell death in SK‐Hep‐1 cells. A) The iron content at 8 and 24 h after exposure to 300 µg mL^−1^ Fe_2_O_3_@DMSA or Fe_2_O_3_@APTS. The data represented mean ± SD. **p < 0.01 and ***p < 0.001 compared with control. ^#^ *p* < 0.05 and ^###^ *p* < 0.001 between the indicated groups. B–E) The mRNA expression levels of typical iron transport systems including TFRC, FTH1, FTL, and FPN in SK‐Hep‐1 cells exposed to 300 µg mL^−1^ Fe_2_O_3_@DMSA or Fe_2_O_3_@APTS. The data represented mean ± SD. *p < 0.05, **p < 0.01, and ***p < 0.001 compared with control. ^##^ *p* < 0.01 and ^###^ *p* < 0.001 between the indicated groups. F) Relative cell proliferation rate of SK‐Hep‐1 cells exposed to gradient concentrations of Fe_2_O_3_@DMSA or Fe_2_O_3_@APTS. The data represented mean ± SD. G,H) Apoptosis and necrosis of SK‐Hep‐1 cells exposed to 20, 200, or 300 µg mL^−1^ Fe_2_O_3_@DMSA or Fe_2_O_3_@APTS. I) Intracellular ROS production of SK‐Hep‐1 cells exposed to 300 µg mL^−1^ Fe_2_O_3_@DMSA or Fe_2_O_3_@APTS with or without NAC for 24 h. The data represented mean ± SD. ***p < 0.001 compared with control. ^###^ *p* < 0.001 between the indicated groups. DMSA denotes Fe_2_O_3_@DMSA. APTS denotes Fe_2_O_3_@APTS.

To further illustrate the mechanism of the enhanced cytotoxicity, hepatoma cells treated with Fe_2_O_3_@DMSA and Fe_2_O_3_@APTS were stained with Annexin V/propidium iodide (PI) and detected by flow cytometry. As illustrated in Figure 2G,H, the levels of apoptosis and necrosis for SK‐Hep‐1 cells were markedly enhanced with increasing concentrations of Fe_2_O_3_@DMSA. Particularly at the concentration of 300 µg mL^−1^, Fe_2_O_3_@DMSA significantly increased the proportion of apoptosis than that of control (DMSA 9.897% ± 0.2650 vs Control 3.318% ± 0.4044, *p* < 0.0001), and also accordingly increased the proportion of necrosis (DMSA 6.813% ± 0.8830 vs Control 2.453% ± 0.2178, *p* = 0.0011). However, cells treated with Fe_2_O_3_@APTS presented only a slight increased amount of apoptosis even at high concentration (300 µg mL^−1^) (APTS 4.906% ± 0.0901 vs Control 3.318% ± 0.4044, *p* = 0.0027), but negligible necrosis (APTS 2.870% ± 0.1480 vs Control 2.453% ± 0.2178, *p* = 0.0519). Similarly, Fe_2_O_3_@DMSA and Fe_2_O_3_@APTS generated similar tendency of cytotoxicity, LDH release, and induced apoptosis and necrosis in HepG2 cells (Figure S3, Supporting Information). These results indicated that Fe_2_O_3_@DMSA had more potent cytotoxicity than Fe_2_O_3_@APTS, which mainly realized through cellular apoptosis and necrosis.

To address whether the cytostatic ability of Fe_2_O_3_@DMSA and Fe_2_O_3_@APTS was HCC specific, normal hepatocytes HL‐7702 were treated with Fe_2_O_3_@DMSA and Fe_2_O_3_@APTS, respectively, with the same treatment strategy as hepatoma cells mentioned above. The relative proliferation rate of HL‐7702 cells both remained almost constant (>85%) with the treatment of Fe_2_O_3_@DMSA and Fe_2_O_3_@APTS below 500 µg mL^−1^ and decreased slowly with the increase of treated doses (Figure S4, Supporting Information). In addition, Fe_2_O_3_@DMSA displayed a little stronger cytotoxic effect on HL‐7702 cells with an IC_50_ of ≈2854.0 µg mL^−1^ compared with Fe_2_O_3_@APTS with an IC_50_ of ≈5441.0 µg mL^−1^. Apparently, Fe_2_O_3_@DMSA and Fe_2_O_3_@APTS possessed a much higher selectivity and antitumor activity in hepatoma cells.

It has been demonstrated that excessive intracellular iron accumulation resulted in severe cellular dysfunction by promoting the production of ROS.^[^ ^14^ ^]^ In this study, we hypothesized that increased ROS would induce the disruptions of cellular Fe_2_O_3_@DMSA metabolism. We first detected ROS level in Fe_2_O_3_@DMSA‐ and Fe_2_O_3_@APTS‐treated hepatoma cells and found that the intracellular ROS levels were remarkably enhanced after Fe_2_O_3_@DMSA treatment for 24 h (FC = 1.902, *p* < 0.0001), but were largely abolished by *N*‐acetyl cysteine (NAC) (DMSA 1.902 ± 0.0497 vs DMSA+NAC 1.265 ± 0.0187, *p* < 0.0001) (Figure 2I), a general membrane‐permeable ROS scavenger.^[^ ^15^ ^]^ Likewise, Fe_2_O_3_@APTS treatment significantly promoted the ROS accumulation but with a lower level compared with Fe_2_O_3_@DMSA treatment (APTS 1.619 ± 0.0482 vs DMSA 1.902 ± 0.0497, *p* = 0.0021). However, this increased ROS induced by Fe_2_O_3_@APTS was not affected by NAC pretreatment (Figure 2I), which might be due to the fact that ROS flux in Fe_2_O_3_@APTS‐treated cells was below the toxic threshold and the redox balance was still maintained.^[^ ^16^ ^]^ Our results indicated that Fe_2_O_3_@DMSA had the ability to induce a sufficiently high intracellular iron accumulation and then gave rise to the generation of excess ROS in hepatoma cells.

Autophagy is a well‐known response to cellular injury and was highly associated with ROS generation.^[^ ^17^, ^18^, ^19^ ^]^ To explore whether autophagy was involved in ROS overload after Fe_2_O_3_@DMSA and Fe_2_O_3_@APTS treatment, SK‐Hep‐1 cells were transfected with adenovirus carrying eGFP‐mRFP‐LC3 to study the assembly of autophagosomes.^[^ ^20^, ^21^ ^]^ Confocal microscopy images showed that Fe_2_O_3_@DMSA was able to increase early autophagosomes formation (yellow dots, before fusion), while Fe_2_O_3_@APTS induced an increase in the number of both autophagosomes and autolysosomes (red dot, after fusion with the lysosome) (**Figure** **3**A). To further discriminate whether the autophagosomes’ accumulation was caused by the induction of autophagic flux or the interdiction of autophagosomes’ degradation, we detected the cellular levels of autophagic markers LC3 and P62 in SK‐Hep‐1 cells using western blot analysis. As shown in Figure 3B, Fe_2_O_3_@DMSA treatment resulted in a significant increase of cellular LC3‐II and accumulation of P62, indicating that autophagic flux was impaired, whereas Fe_2_O_3_@APTS treatment induced a slight accumulation of LC3‐II and P62. The messenger ribonucleic acid (mRNA) expression levels of autophagy markers that were demonstrated to be required and directly involved in the formation of autophagosomes (LC3B, P62, Beclin1, Atg2A, Atg16L1, Atg17, and Atg18)^[^ ^22^ ^]^ were also examined in Fe_2_O_3_@DMSA‐ and Fe_2_O_3_@APTS‐treated SK‐Hep‐1 cells. The quantitative real‐time polymerase chain reaction (PCR) (q‐PCR) results showed that the autophagy‐formation‐related genes were obviously activated both in Fe_2_O_3_@DMSA‐ and Fe_2_O_3_@APTS‐treated cells, and the activation ability of Fe_2_O_3_@DMSA was much higher than Fe_2_O_3_@APTS (Figure S5, Supporting Information).

**Figure 3:**
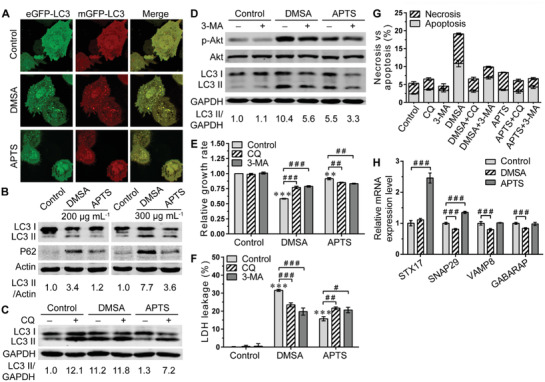
Fe_2_O_3_@DMSA differed from Fe_2_O_3_@APTS in their ability to induce autophagy and apoptosis in SK‐Hep‐1 cells. A) Confocal microscopy assay for SK‐Hep‐1 cells transfected with adenovirus carrying eGFP‐mRFP‐LC3. B) The expressions of LC3 and P62 protein in SK‐Hep‐1 cells exposed to Fe_2_O_3_@DMSA or Fe_2_O_3_@APTS. C) The expressions of LC3 protein in SK‐Hep‐1 cells exposed to 300 µg mL^−1^ Fe_2_O_3_@DMSA or Fe_2_O_3_@APTS with or without CQ. D) The expressions of p‐Akt, Akt, and LC3 protein in SK‐Hep‐1 cells exposed to 300 µg mL^−1^ Fe_2_O_3_@DMSA or Fe_2_O_3_@APTS with or without 3‐MA. E) Relative cell growth rate of SK‐Hep‐1 cells exposed to 300 µg mL^−1^ Fe_2_O_3_@DMSA or Fe_2_O_3_@APTS with or without CQ or 3‐MA. The data represented mean ± SD. **p < 0.01 and ***p < 0.001 compared with control. ^##^ *p* < 0.01 and ^###^ *p* < 0.001 between the indicated groups. F) LDH release of SK‐Hep‐1 cells exposed to 300 µg mL^−1^ Fe_2_O_3_@DMSA or Fe_2_O_3_@APTS with or without CQ or 3‐MA. The data represented mean ± SD. ***p < 0.001 compared with control. ^#^ *p* < 0.05, ^##^ *p* < 0.01, and ^###^ *p* < 0.001 between the indicated groups. G) Apoptosis and necrosis of SK‐Hep‐1 cells exposed to 300 µg mL^−1^ Fe_2_O_3_@DMSA or Fe_2_O_3_@APTS with or without CQ or 3‐MA. The data represented mean ± SD. H) The mRNA expression levels of typical autophagosome–lysosome fusion markers, including STX17, SNAP29, VAMP8, and GABARAP, in SK‐Hep‐1 cells exposed to 300 µg mL^−1^ Fe_2_O_3_@DMSA or Fe_2_O_3_@APTS were detected by q‐PCR. The data represented mean ± SD. ^###^ *p* < 0.001 between the indicated groups. DMSA denotes Fe_2_O_3_@DMSA. APTS denotes Fe_2_O_3_@APTS.

Chloroquine acts as an autophagy inhibitor of the fusion of autophagosomes and lysosomes, via raising the pH in the lumen of lysosomes and/or autolysosomes, leading to autophagic degradation blockage and accumulation of mature autophagic vacuoles.^[^ ^23^, ^24^ ^]^ Noteworthy, CQ treatment induced practically no further accumulation of LC3‐II in Fe_2_O_3_@DMSA groups, but resulted in a visually identical accumulation of LC3‐II complexes in Fe_2_O_3_@APTS groups (Figure 3C). 3‐methyladenine (3‐MA) is another autophagy inhibitor that works on the PI3K‐Akt signaling pathway to block autophagosomes formation.^[^ ^25^ ^]^ It was capable of decreasing the phosphorylation of Akt and the autophagy induced by Fe_2_O_3_@DMSA and Fe_2_O_3_@APTS treatment (Figure 3D). Collectively, these results strongly indicated that Fe_2_O_3_@DMSA induced cellular autophagic activation and autophagosomes accumulation in SK‐Hep‐1 cells, whereas Fe_2_O_3_@APTS accelerated the autophagic process. These results were consistent with the TEM data that early autophagic vesicles with double membrane were observed in Fe_2_O_3_@DMSA‐treated cells, while degradative autophagic vesicles with single membrane were present only in Fe_2_O_3_@APTS‐treated cells (Figure 1I,J).

After verification the different abilities of Fe_2_O_3_@DMSA and Fe_2_O_3_@APTS in upregulating cellular autophagic level, we continued to investigate if the autophagy induced by Fe_2_O_3_@DMSA and Fe_2_O_3_@APTS could contribute to their cytotoxicity efficacy. Both CQ and 3‐MA treatment presented an enhanced cell proliferation rate (DMSA 0.5837 ± 0.0065 vs DMSA+CQ 0.7704 ± 0.0198, *p* = 0.0001; DMSA 0.5837 ± 0.0065 vs DMSA+3‐MA 0.7883 ± 0.0121, *p* < 0.0001) and decreased LDH leakage (DMSA 31.53 ± 0.5886 vs DMSA+CQ 23.48 ± 1.116, *p* = 0.0004; DMSA 31.53 ± 0.5886 vs DMSA+3‐MA 19.74 ± 1.979, *p* = 0.0006) in Fe_2_O_3_@DMSA‐treated cells, but resulted in a significant drop of cell proliferation rate (APTS 0.9157 ± 0.0178 vs APTS +CQ 0.8509 ± 0.0070, *p* = 0.0042; APTS 0.9157 ± 0.0178 vs APTS +3‐MA 0.8346 ± 0.0062, *p* = 0.0017) and a marked raise of LDH leakage (APTS 15.75 ± 1.153 vs APTS +CQ 21.59 ± 0.7507, *p* = 0.0018; APTS 15.75 ± 1.153 vs APTS +3‐MA 20.59 ± 1.541, *p* = 0.0121) in Fe_2_O_3_@APTS groups (Figure 3E,F). To further evaluate the enhanced cytotoxicity, cells with different treatments were stained with Annexin V/fluorescein isothiocyanate (FITC) and PI, and then processed by flow cytometry. Results showed that (Figure 3G; Figure S6, Supporting Information) both Fe_2_O_3_@DMSA and Fe_2_O_3_@APTS clearly enhanced the apoptotic to necrotic cell ratio, but Fe_2_O_3_@DMSA‐treated cells presented higher apoptotic (DMSA 10.87 ± 0.9152 vs APTS 3.497 ± 0.1767, *p* = 0.0002) and necrotic (DMSA 8.263 ± 0.2367 vs APTS 4.870 ± 0.0781, *p* < 0.0001) levels compared with cells treated with Fe_2_O_3_@APTS. However, both CQ and 3‐MA co‐culture led to a significant reduction of apoptosis (DMSA 10.87 ± 0.9152 vs DMSA+CQ 3.165 ± 0.3451, *p* = 0.0002; DMSA 10.87 ± 0.9152 vs DMSA+3‐MA 6.883 ± 0.3609, *p* = 0.0022) and necrosis (DMSA 8.263 ± 0.2367 vs DMSA+CQ 3.400 ± 0.4571, *p* < 0.0001; DMSA 8.263 ± 0.2367 vs DMSA+3‐MA 3.013 ± 0.1950, *p* < 0.0001) induced by Fe_2_O_3_@DMSA, but no significant difference was found in Fe_2_O_3_@APTS‐treated cells, indicating that Fe_2_O_3_@DMSA improved cytotoxicity depending on the activation of autophagy pathway.

It has been reported that STX17 interacts with SNAP29 and the lysosomal VAMP8 to promote autophagosome–lysosome fusion.^[^ ^26^, ^27^ ^]^ Depletion of STX17 causes accumulation of autophagosomes without degradation.^[^ ^26^ ^]^ Moreover, knockdown of GABARAP creates defective autophagosomes and impairs the degradation of LC3 and P62, owing to defective autophagosomal fusion with the lysosome.^[^ ^28^, ^29^ ^]^ To provide insight into the contributions of autophagy to cytotoxicity/survival efficacy of Fe_2_O_3_@DMSA and Fe_2_O_3_@ATPS, we further detected the expression of typical autophagosome–lysosome fusion markers (STX17, SNAP29, VAMP8, and GABARAP) in Fe_2_O_3_@DMSA‐ and Fe_2_O_3_@APTS‐treated cells. The q‐PCR results showed that the expression levels of SNAP29 (FC = 0.813, *p* < 0.0001), VAMP8 (FC = 0.797, *p* = 0.0009), and GABARAP (FC = 0.837, *p* = 0.0001) were significantly decreased in Fe_2_O_3_@DMSA‐treated cells. In contrast, STX17 (FC = 2.453, *p* < 0.0001) and SNAP29 (FC = 1.351, *p* < 0.0001) were remarkably increased in Fe_2_O_3_@APTS‐treated cells (Figure 3H), indicating that Fe_2_O_3_@DMSA promoted cell apoptosis and necrosis through inducing cellular autophagic activation and impairing autophagosome–lysosome fusion, whereas Fe_2_O_3_@APTS alleviated cytotoxicity via accelerating the autophagic process.

To confirm this apoptosis‐promoting role of enhanced autophagy in Fe_2_O_3_@DMSA‐induced cell proliferation inhibition, we performed knockdown of two different autophagy‐essential genes (Atg5 and Atg7) with small interfering ribonucleic acid (siRNA). Transfection of cultured SK‐Hep‐1 cells with Atg5‐ or Atg7‐targeting siRNA resulted in large reductions in the levels of the corresponding proteins (Figure S7A, Supporting Information). As expected, downregulation of both Atg5 and Atg7 extremely ameliorated Fe_2_O_3_@DMSA‐induced cell proliferation inhibition and LDH leakage, but augmented Fe_2_O_3_@APTS‐induced cell proliferation inhibition and LDH leakage (Figure S7B,C, Supporting Information). Moreover, suppression of Atg5 obviously decreased Fe_2_O_3_@DMSA‐ and Fe_2_O_3_@APTS‐induced autophagy as shown by a decrease in LC3‐II formation and the inhibition of P62 accumulation (Figure S7D, Supporting Information). All together, these data further confirmed our hypothesis that blocking autophagy using genetic inhibition could decrease the autophagy‐promoted cytotoxicity of Fe_2_O_3_@DMSA, but enhance the cell cytostatic effect of Fe_2_O_3_@APTS.

To determine whether enhanced ROS levels are required for Fe_2_O_3_@DMSA and Fe_2_O_3_@APTS to activate autophagy, we detected the expression levels of LC3 protein. Our results showed that NAC effectively inhibited the induction of LC3‐II in Fe_2_O_3_@DMSA‐treated cells, but failed to decrease LC3‐II levels in the present of Fe_2_O_3_@APTS (**Figure** **4**A), suggesting that Fe_2_O_3_@DMSA treatment leads to robust ROS accumulation, thereby activating autophagy but causing severe damage to autophagic flux, whereas Fe_2_O_3_@APTS‐induced moderate and persisted ROS increase may serve as a sufficient signal to trigger autophagy.^[^ ^30^ ^]^ Consistently, cell proliferation inhibition (DMSA 0.5837 ± 0.0065 vs DMSA+NAC 0.8276 ± 0.0077, *p* < 0.0001) as well as cellular apoptosis (DMSA 10.87 ± 0.9152 vs DMSA+NAC 6.473 ± 0.3707, *p* = 0.0014) and necrosis (DMSA 8.263 ± 0.2367 vs DMSA+NAC 4.943 ± 0.3066, *p* = 0.0001) induced by Fe_2_O_3_@DMSA were largely blocked by NAC. However, these effects were insensitive during Fe_2_O_3_@APTS treatment (Figure 4B–D).

**Figure 4:**
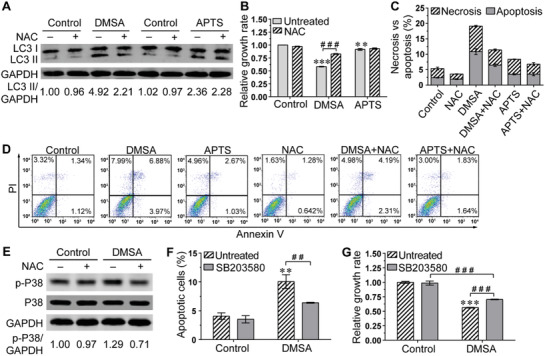
Excess ROS‐triggered autophagic cell death in Fe_2_O_3_@DMSA‐treated SK‐Hep‐1 cells through P38 MAPK signaling pathways. A) The expressions of LC3 protein in SK‐Hep‐1 cells exposed to Fe_2_O_3_@DMSA or Fe_2_O_3_@APTS with or without NAC. B) Relative cell growth rate of SK‐Hep‐1 cells exposed to Fe_2_O_3_@DMSA or Fe_2_O_3_@APTS with or without NAC. The data represented mean ± SD. **p < 0.01 and ***p < 0.001 compared with control. ^###^ *p* < 0.001 between the indicated groups. C,D) Apoptosis and necrosis of SK‐Hep‐1 cells exposed to Fe_2_O_3_@DMSA or Fe_2_O_3_@APTS with or without NAC. The data represented mean ± SD. E) The expressions of p‐P38 and P38 protein in SK‐Hep‐1 cells exposed to Fe_2_O_3_@DMSA with or without NAC. F) Apoptosis of SK‐Hep‐1 cells exposed to Fe_2_O_3_@DMSA with or without SB203580. The data represented mean ± SD. **p < 0.01 compared with control. ^##^ *p* < 0.01 between the indicated groups. G) Relative cell growth rate of SK‐Hep‐1 cells exposed to Fe_2_O_3_@DMSA with or without SB203580. The data represented mean ± SD. ***p < 0.001 compared with control. ^###^ *p* < 0.001 between the indicated groups. DMSA denotes 300 µg mL^−1^ Fe_2_O_3_@DMSA. APTS denotes 300 µg mL^−1^ Fe_2_O_3_@APTS.

To further investigate the molecular mechanism underlying the differential ROS‐inducing autophagy activity of Fe_2_O_3_@DMSA versus Fe_2_O_3_@APTS, we still detected the expression level of p‐P38, acted as proapoptotic and necrotic stimuli,^[^ ^31^, ^32^ ^]^ and found that Fe_2_O_3_@DMSA induced elevation of p‐P38, which was significantly inhibited by NAC, while no such result was observed in Fe_2_O_3_@APTS treatment (Figure 4E). SB203580, a highly specific and potent inhibitor of P38 mitogen‐activated protein (MAP) kinase,^[^ ^33^, ^34^ ^]^ effectively reduced Fe_2_O_3_@DMSA‐induced cellular apoptosis (DMSA 10.06 ± 1.184 vs DMSA+SB203580 6.385 ± 0.0829, *p* = 0.0059) and proliferation inhibition (DMSA 0.5626 ± 0.0065 vs DMSA+SB203580 0.7043 ± 0.0064, *p* < 0.0001) (Figure 4F,G), but did not disrupt the effect of Fe_2_O_3_@APTS (Figure S8, Supporting Information). Furthermore, western blot results showed that P38 inhibitor BS203580 did not further affect the level of autophagy induced by Fe_2_O_3_@DMSA (Figure S9, Supporting Information). Collectively, the above results demonstrated that Fe_2_O_3_@DMSA‐induced autophagic cell death, through excess ROS production, was dependent on the function of P38 mitogen‐activated protein kinase (MAPK) signaling pathways.

Accumulating testimonies have documented that the induced autophagy could have either prodeath or prosurvival impact on the cancer cell.^[^ ^35^ ^]^ Notably, as shown in Figure 3, Fe_2_O_3_@APTS alleviated cytotoxicity via accelerating the autophagic process. MAPK/extracellular signal‐regulated kinase (ERK) pathways are often involved in the regulation of cell survival.^[^ ^31^, ^36^ ^]^ We thus detected the corresponding proteins and their activation situation. As shown in **Figure** **5**A, Fe_2_O_3_@APTS treatment led to an increase of p‐ERK, which was not affected in the presence of Fe_2_O_3_@DMSA. In agreement, autophagy inhibition with CQ and Atg5 siRNA dramatically decreased the enhanced level of p‐ERK induced by Fe_2_O_3_@APTS (Figure 5B,C). On the other hand, the addition of U0126, a highly selective inhibitor of ERK kinase,^[^ ^37^, ^38^, ^39^ ^]^ significantly suppressed the phosphorylation of ERK elicited by Fe_2_O_3_@APTS (Figure 5D), but caused a further increase of cellular apoptosis (APTS 3.433 ± 0.0681 vs APTS +U0126 8.811 ± 0.8489, *p* = 0.0004) and necrosis (APTS 4.960 ± 0.2339 vs APTS +U0126 17.73 ± 0.8963, *p* < 0.0001) as well as the reduction of cell proliferation (APTS 0.897 ± 0.0102 vs APTS +U0126 0.749 ± 0.0110, *p* < 0.0001) (Figure 5E–G). However, U0126 had little impact on Fe_2_O_3_@DMSA‐induced cellular proliferation inhibition (Figure S10, Supporting Information). Meanwhile, the upregulated LC3‐II protein induced by Fe_2_O_3_@APTS had no visible difference in the present of ERK inhibitor U0126 (Figure S11, Supporting Information). These data in combination with the results in Figure 3 implied that p‐ERK was the downstream of autophagy and necessary for Fe_2_O_3_@APTS to trigger cytoprotective autophagy, and thus alleviating cytotoxicity.

**Figure 5:**
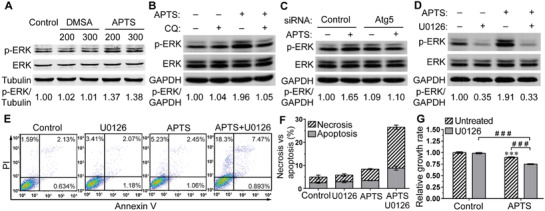
Autophagy induced by APTS promoted cell survival in SK‐Hep‐1 cells. A) The expressions of p‐ERK and ERK protein in SK‐Hep‐1 cells exposed to Fe_2_O_3_@DMSA or Fe_2_O_3_@APTS, at 200 and 300 µg mL^−1^. B) The expressions of p‐ERK and ERK protein in SK‐Hep‐1 cells exposed to 300 µg mL^−1^ Fe_2_O_3_@APTS with or without CQ. C) The expressions of p‐ERK and ERK protein in Atg5 depletion cells exposed to 300 µg mL^−1^ Fe_2_O_3_@APTS. D) The expressions of p‐ERK and ERK proteins in SK‐Hep‐1 cells exposed to 300 µg mL^−1^ Fe_2_O_3_@APTS with or without U0126. E,F) Apoptosis and necrosis in SK‐Hep‐1 cells exposed to 300 µg mL^−1^ Fe_2_O_3_@APTS with or without U0126. G) Relative cell growth rate of SK‐Hep‐1 cells exposed to 300 µg mL^−1^ Fe_2_O_3_@APTS with or without U0126. The data represented mean ± SD. ***p < 0.001 compared with control. ^###^ *p* < 0.001 between the indicated groups. DMSA denotes Fe_2_O_3_@DMSA. APTS denotes Fe_2_O_3_@APTS.

Encouraged by the results from the in vitro studies, we established two subcutaneous xenograft nude mouse models to explore the in vivo antitumor efficacy of Fe_2_O_3_@DMSA and Fe_2_O_3_@APTS. In the first model, we subcutaneously implanted 2 × 10^6^ SK‐Hep‐1 cells into the right hind limbs of nude mice, with or without addition of NPs. It could be observed that the tumor volumes in Fe_2_O_3_@DMSA shrunk significantly compared with those in controls (DMSA 146.7 ± 29.54 mm^3^ vs Control 297.7 ± 43.46 mm^3^, *p* = 0.0002) at the endpoint, while Fe_2_O_3_@APTS had slight therapeutic efficacy (APTS 230.8 ± 51.53 mm^3^ vs Control 297.7 ± 43.46 mm^3^, *p* = 0.0581) (**Figure** **6**A). Furthermore, even at the endpoint of the tumor growth curve, the isolated tumor volume and tumor weight in the Fe_2_O_3_@DMSA group were much decreased than those of the control group (Figure 6C–E). It was noteworthy that the Fe_2_O_3_@DMSA group exhibited a much remarkable decrease in tumor volume (DMSA 101.6 ± 7.97 mm^3^ vs APTS 163.6 ± 17.98 mm^3^, *p* = 0.0007) and tumor weight (DMSA 0.0853 ± 0.015 g vs APTS 0.1377 ± 0.025 g, *p* = 0.0113) compared with that of the Fe_2_O_3_@APTS group. In the second model, SK‐Hep‐1 cells were subcutaneously injected into the right hind limbs of nude mice. Then, the tumor‐bearing mice were tail vein injected with Fe_2_O_3_@DMSA, Fe_2_O_3_@APTS, or 0.9% saline, respectively, once every 3 or 4 days. As expected, there was a remarkable decrease in tumor volume at the endpoint of the tumor growth curve in both Fe_2_O_3_@DMSA and Fe_2_O_3_@APTS groups (Figure 6F,H). This drop of tumor size in the Fe_2_O_3_@DMSA group was much greater than that of the Fe_2_O_3_@APTS group (DMSA 394.8 ± 92.48 mm^3^ vs APTS 612.5 ± 78.30 mm^3^, *p* = 0.0013), demonstrating that Fe_2_O_3_@DMSA was more effective on antitumor than Fe_2_O_3_@APTS.

**Figure 6:**
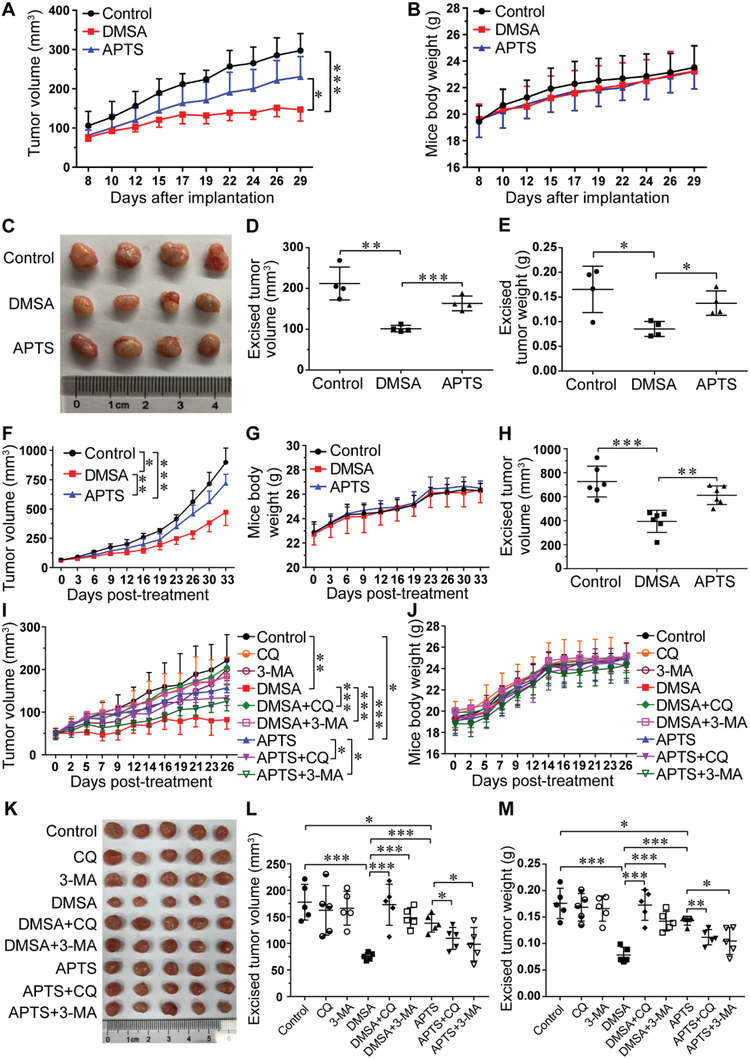
The antitumor efficacy of Fe_2_O_3_@DMSA and Fe_2_O_3_@APTS. A–E) In the first model, SK‐Hep‐1 cells with or without addition of NPs were subcutaneously co‐implanted into the right hind limbs of nude mice. F–M) In the second model, SK‐Hep‐1 cells were subcutaneously injected into the right hind limbs of nude mice. When the tumor volume reached ≈50 mm^3^, the tumor‐bearing mice were tail vein injected with indicated formulation for over 11 times. Dosing used: Fe_2_O_3_@DMSA, 40 µg; Fe_2_O_3_@APTS, 40 µg; CQ, 10 mg kg^−1^; 3‐MA, 80 µmol kg^−1^. A,B) The tumor volume and body weight changes of mice during the 22 day therapeutic period (*n* = 4). C) Photographs of the tumors excised on day 29 (*n* = 4). D,E) The excised tumor volume and tumor weight of mice on day 29 (*n* = 4). F,G) The tumor volume and body weight changes of mice during the 33 day therapeutic period (*n* = 6). H) The excised tumor volume of mice at the endpoint of the therapeutic period (*n* = 6). I,J) The tumor volume and body weight changes of mice during the 26 day therapeutic period (*n* = 5). K) Photographs of the tumors excised at the endpoint of the therapeutic period (*n* = 5). L,M) The excised tumor volume and tumor weight of mice at the endpoint of the therapeutic period (*n* = 5). All data were shown as mean ± SD. *p < 0.05, **p < 0.01, and ***p < 0.001 between the indicated groups. DMSA denotes Fe_2_O_3_@DMSA. APTS denotes Fe_2_O_3_@APTS.

To assess the involvement of autophagy in Fe_2_O_3_@DMSA‐ and Fe_2_O_3_@APTS‐mediated in vivo antitumor efficacy, autophagy inhibitors CQ and 3‐MA were used in antitumor trials. As expected, both Fe_2_O_3_@DMSA and Fe_2_O_3_@APTS greatly inhibited tumor growth, with Fe_2_O_3_@DMSA exhibiting a significantly better effect (DMSA 82.4 ± 22.34 mm^3^ vs APTS 157.2 ± 11.54 mm^3^, *p* = 0.00002), as anticipated by the change in tumor volume (Figure 6I). However, co‐treatment with CQ or 3‐MA obviously suppressed the antitumor effect of Fe_2_O_3_@DMSA (DMSA 82.4 ± 22.34 mm^3^ vs DMSA+CQ 204.8 ± 39.2 mm^3^, *p* = 0.0003; DMSA 82.4 ± 22.34 mm^3^ vs DMSA+3‐MA 184.5 ± 20.90 mm^3^, *p* < 0.0001). Importantly, compared with Fe_2_O_3_@APTS treatment alone, adding CQ or 3‐MA dramatically reduced the tumor volume (APTS 157.2 ± 11.54 mm^3^ vs APTS+CQ 134.0 ± 18.87 mm^3^, *p* = 0.0467; APTS 157.2 ± 11.54 mm^3^ vs APTS+3‐MA 125.4 ± 22.64 mm^3^, *p* = 0.0233). At the end of the experimental period, the tumors were harvested and photographed. Concordantly, the isolated tumor volume as well as tumor weight agreed well with the tumor volume growth curves (Figure 6K–M). These results were in consistent with the in vitro data, indicating that Fe_2_O_3_@DMSA exhibited a higher antitumor efficacy than Fe_2_O_3_@APTS by activating the autophagy pathway, whereas Fe_2_O_3_@APTS needed to be used in combination with autophagy inhibitors to achieve better antitumor effect, which fully proved that Fe_2_O_3_@DMSA had excellent antitumor effect without adding any autophagy inhibitors.

To evaluate the safety profile of these exposures of NPs, the mice's body weights were tracked and recorded through the whole process of in vivo antitumor study. In the both models, compared with the control group, mice in both Fe_2_O_3_@DMSA and Fe_2_O_3_@APTS groups exhibited similar constant increase in bodyweight (Figure 6B,G). Additionally, there was no significant difference in mice's body weight during the course of antitumor therapy among co‐treatment with CQ or 3‐MA (Figure 6J). Further analysis of the serum biochemistry, including hepatic function markers (alanine aminotransferase (ALT) and aspartate aminotransferase (AST)) and renal function markers (blood urea nitrogen (BUN) and creatinine (CREA)), was examined, and no significant difference was detected (Figure S12A–C, Supporting Information). In addition, from the hematoxylin and eosin (H&E) stained slices, no apparent lesions, inflammation, or morphological change was observed in major organs in each formulation group (Figure S12D, Supporting Information). Together, these results provide evidence that Fe_2_O_3_@DMSA is safe and nontoxic in vivo for antitumor applications.

Autophagy intervention has been proved to be a promising therapeutic strategy for cancer therapy.^[^ ^40^ ^]^ Therefore, various treatment methods based on autophagy intervention have attracted great attention. Previous studies have found that most nanoparticles have the ability to induce autophagic activation of tumor cells; thus, the combined treatment of nanoparticles with autophagy inhibitors, such as CQ or HCQ, could enhance their antitumor efficiency.^[^ ^7^, ^8^ ^]^ However, these treatment methods still remain great challenges. First, they could not overcome the major shortcoming of nonselective in vivo distribution of clinical CQ or HCQ administration. Second, the autophagy inhibitors could induce a serious retinopathy.^[^ ^12^, ^13^ ^]^ These shortcomings greatly limit the application of “nanoparticles combined with autophagy inhibitors” in the clinical treatment of cancer.

Differ from previous investigations, our present research elaborated a new discovery and demonstrated that Fe_2_O_3_@DMSA specifically triggered intracellular iron‐retention‐induced sustained ROS generation, which not only dramatically induced cellular autophagic activation, but also, more importantly, extensively impaired the autophagic flux by inhibiting the fusion of autophagosomes and lysosomes, thus, significantly promoting autophagic death of tumor cells. Furthermore, this tumoricidal autophagy of Fe_2_O_3_@DMSA was further effectively verified in xenograft mouse models without adding any exogenous autophagy inhibitors. However, contrary to Fe_2_O_3_@DMSA, most nanoparticles currently used, such as IONPs and CuPd tetrapods,^[^ ^7^, ^8^ ^]^ as well as the Fe_2_O_3_@APTS verified in our present study, significantly activated autophagy and promoted the fusion of autophagosomes and lysosomes, and then accelerated the autophagic process, which led to poor antitumor efficiency. Collectively, in this study, a novel feature of Fe_2_O_3_@DMSA design is that it inhibits the fusion of autophagosomes and lysosomes that cause a significant suppression of cancer growth, and also it provides novel insight into the design and application of nanoparticles for cancer therapy.

In conclusion, we have demonstrated that Fe_2_O_3_@DMSA induces autophagy activation and impairs autophagy flux in tumor cells, so as to exert tumoricidal autophagy for cancer therapy without adding exogenous autophagy intervention agents. Furthermore, the tumoricidal autophagy of Fe_2_O_3_@DMSA not only overcomes the main challenge of nonselectivity in vivo distribution of autophagy intervention agents, but also successfully avoids the serious side effects of autophagic chemical intervention agents. Thus, this Fe_2_O_3_@DMSA antitumor system provides us a potential strategy for cancer therapy and is beneficial for the development of IONPs for biomedical applications.

## Experimental Section

##### Chemicals and Materials

Carboxy‐functional Fe_2_O_3_ core nanoparticles (Fe_2_O_3_@DMSA NPs, 10 nm) and amine‐functional Fe_2_O_3_ core nanoparticles (Fe_2_O_3_@APTS NPs, 10 nm) were purchased from Nanjing Nanoeast Biotech Co., Ltd. (Nanjing, Jiangsu, China). Fetal bovine serum (FBS), Dulbecco's modified Eagle medium (DMEM), penicillin, streptomycin, and 0.25% ethylene diamine tetraacetic acid (EDTA) trypsin were purchased from GIBCO, Life Technologies (Grand Island, NY, USA). CQ, 3‐MA, SB203580, and U0126 were purchased from Sigma–Aldrich (St. Louis, MO, USA). Phenylmethanesulfonyl fluoride (PMSF), 2′,7′‐dichlorofluorescin diacetate (DCFH‐DA), *N*‐acetyl‐l‐cysteine (NAC), and lactic dehydrogenase (LDH) cytotoxicity assay kit were purchased from Beyotime Institute of Biotechnology (Nantong, Jiangsu, China). FITC Annexin V Apoptosis Detection Kit was purchased from BD Biosciences (San Diego, CA, USA). Antibodies including P62, p‐Akt (Ser473), Akt, p‐P38 (Thr180/Tyr182), P38, p‐ERK (Thr202/Tyr204), ERK, Atg5, and Atg7 were purchased from Cell Signaling Technology (Danvers, MA, USA). LC3B antibodies were obtained from Novus Biologicals (Littleton, CO, USA). Glyceraldehyde 3‐phosphate dehydrogenase (GAPDH), *β*‐actin, and Tubulin antibodies were purchased from HuaAn Biotechnology (Hangzhou, Zhejiang, China). The IRDye 800CW conjugated secondary antibody was obtained from LI‐COR Biosciences (Lincoln, NE, USA). Mammalian protein extraction (M‐PER) mammalian protein extraction reagent and bicinchoninic acid (BCA) protein assay kit were purchased from Thermo Scientific (Rockford, lL, USA). Complete inhibitor cocktail was purchased from Roche Diagnostics (Mannheim, Germany). Nitrocellulose membrane was purchased from Whatman (Maidstone, Kent, UK).

##### Nanoparticles’ Characterization

The morphology of the nanoparticles was observed using a transmission electron microscope (JEM‐1200EX, JEOL, Ltd., Japan) at an acceleration voltage of 120 kV and an AFM (Dimension Icon, Bruker, USA). The microstructure of the nanoparticles was identified by a field emission scanning electron microscope (S‐4800, Hitachi, Japan). FTIR spectroscopy measurements were performed on an FTIR spectrometer (Nicolet iS10, Thermo Scientific, USA). The zeta potential distribution of the nanoparticles was determined using a Zetasizer Nano ZSP analyzer (Malvern Instruments, Malvern, UK).

##### Cell Culture and Treatment

Hepatoma cell lines SK‐Hep‐1 and HepG2, and normal hepatocytes HL‐7702 were obtained from the Shanghai Cell Bank of China (Shanghai, China). SK‐Hep‐1 and HepG2 cells were grown in DMEM medium containing 4.5 g L^−1^ glucose. HL‐7702 cells were maintained in RPMI 1640 medium. Both culture media contained 100 U mL^−1^ penicillin, 100 µg mL^−1^ streptomycin, and 10% FBS. All cells were maintained in a fully humidified incubator with 5% CO_2_ at 37 °C. Cells were first seeded in plates and incubated for 24 h, and then exposed to nanoparticles for another 8 or 24 h. In some experiments, cells were separately pretreated with CQ (10 × 10^−6^ m), 3‐MA (2 × 10^−3^ m), NAC (400 × 10^−6^ m), SB203580 (10 × 10^−6^ m), and U0126 (5 × 10^−6^ m) for 2 h, then exposed to nanoparticles with indicated inhibitor for another 24 h.

##### Cellular Uptake of Nanoparticles

The flow cytometry was used to measure the cellular uptake of nanoparticles. After treated for 24 h, cells (2 × 10^5^ cells in 12‐well plate) were washed with cold phosphate‐buffered saline (PBS), harvested by trypsinization, and suspended with PBS at the concentration of 1 × 10^6^ cells mL^−1^. Then, the cellular uptake amount was quantitatively determined by flow cytometry (FACSCalibur, BD Biosciences, San Jose, CA, USA).

##### Internalization and Localization of Nanoparticles

Internalization and localization of Fe_2_O_3_@DMSA and Fe_2_O_3_@APTS in cells were observed using TEM. After treated, cells (6 × 10^6^ cells in 10 cm dish) were washed with PBS, scraped, harvested, and fixed in 2.5% glutaraldehyde for 1 day, then post‐fixed in 1% OsO_4_ followed by 2% uranyl acetate. After dehydration, cells were embedded and then cut into slices (80 nm). Each slice was post‐stained with 2% uranyl acetate followed by 0.3% lead citrate, and imaged using TEM (HT7700, Hitachi, Ltd., Japan). Early and degradative autophagosome‐induced by nanoparticles were also observed using TEM.

##### Iron Content Determination

Intracellular iron level was determined using an iron assay kit from Sigma–Aldrich according to the manufacturer's instructions. Briefly, after treated with IONPs, the cells (6 × 10^6^ cells in 10 cm dish) were rapidly homogenized in iron assay buffer, centrifuged at 16 000 × *g* for 10 min at 4 °C to remove insoluble materials. About 100 µL of the supernatant was used to measure absorbance at 593 nm. The concentration of iron was calculated using the standard curve obtained, and the results were normalized to protein concentration of each sample.

##### Transient siRNA Transfection

Transient inhibition of Atg5 and Atg7 was carried out by transferring cells with 50 × 10^−9^ m siRNA or the scrambled siRNA as control (Cell Signaling Technology, Danvers, MA, USA) for 24 h using Lipofectamine 2000 (Invitrogen) according to the manufacturer's instructions. Then the cells were harvested and re‐seeded for further experimental utilizations.

##### Adenovirus Infection and Identification of Autophagy

After cells (3 × 10^5^ cells in 35 mm dish) seeded overnight, the adenovirus carrying eGFP‐mRFP‐LC3 was infected into the cells for 6 h. Then, the cells were washed with PBS and maintained with fresh medium for another 18 h, followed by nanoparticles’ exposure. As soon as the treatment was completed, the eGFP‐mRFP‐LC3 dots were detected using a confocal fluorescence microscope (Leica TCS SP8 X, Leica Corporation, Germany).

##### Cell Proliferation Assay

The cytotoxicity of Fe_2_O_3_@DMSA and Fe_2_O_3_@APTS was performed with the cell proliferation assay. As soon as the treatment was completed, cells (2 × 10^5^ cells in 12‐well plate) were trypsinized and counted by a cell coulter (Beckman counter Z2, Hialeah, FL, USA). The relative cell proliferation rate was calculated by normalizing the number of nanoparticles or chemical‐treated cells to nonexposed control cells.

##### Cellular Membrane Damage Assay

The cellular membrane damage assay was performed using the LDH assay kit as previously described.^[^ ^41^ ^]^ Cells were treated with Fe_2_O_3_@DMSA or Fe_2_O_3_@APTS for 24 h.

##### Apoptosis and Necrosis Assays

Apoptosis and necrosis were detected using the FITC Annexin V Apoptosis Detection Kit and measured with flow cytometer as previously described.^[^ ^41^ ^]^ The perception of apoptotic and necrotic cells was analyzed with FlowJo software. Cells were treated with Fe_2_O_3_@DMSA or Fe_2_O_3_@APTS for 24 h.

##### Western Blot Assay

The treated cells (1.5 × 10^6^ cells in 6 cm dish) were washed three times with cold PBS and lysed using M‐PER mammalian protein extraction reagent supplemented with complete inhibitor cocktail and PMSF. The protein concentration in the supernatant fraction was determined by the pierce BCA protein assay kit. Equal amounts of protein were separated by sodium dodecyl sulfate‐polyacrylamide gel electrophoresis (SDS‐PAGE) and transferred onto an nitrocellulose (NC) membrane. The membranes were blocked with 5% bovine serum albumin (BSA) in Tris‐buffered saline (TBS) for 1 h, followed by primary antibodies’ incubation as indicated at 4 °C overnight. After incubation with secondary antibodies, the protein bands were visualized using an odyssey infrared imaging system (LI‐COR Biosciences) and quantified with odyssey application software. GAPDH, *β*‐actin, and Tubulin were used for the loading control.

##### ROS Assay

Intracellular ROS levels were detected using DCFH‐DA and analyzed by flow cytometer as previously described.^[^ ^41^ ^]^ Cells were treated with Fe_2_O_3_@DMSA or Fe_2_O_3_@APTS for 24 h.

##### RNA Extraction, Reverse Transcription, and q‐PCR

Total RNA was extracted from the treated cells with TRIzol according to the manufacturer's instruction (Ambion, Life Technologies). The RNA concentrations were determined by a NanoDrop 2000 spectrophotometer. Then, 1.0 µg of RNA for each sample was reverse‐transcribed using the PrimeScript RT Reagent Kit with gDNA Eraser according to the manufacturer's instructions (TaKaRa). The q‐PCR was conducted using TB Green Premix Ex Taq II according to the manufacturer's instructions (TaKaRa). The relative gene expression was calculated using the ΔΔ*Ct* method, and GAPDH was used as the internal control. The primers used in the PCR reactions were shown in Table S1 (Supporting Information).

##### Animals

BALB/c nude mice (male, 5 weeks, 16–18 g) were purchased from Shanghai SLAC Laboratory Animals Co., Ltd. (Shanghai, China). The mice were maintained at an animal facility under specific pathogen‐free conditions. All animal care procedures and surgical interventions were approved by Ren Ji Hospital Ethics Committee. All animal experiments were carried out in accordance with the guidelines of China Animal Welfare Legislation. All efforts were made to minimize suffering.

##### In Vivo Models

Two subcutaneous xenograft nude mouse models were established. In the first model, 2 × 10^6^ SK‐Hep‐1 cells were subcutaneously implanted into the right hind limbs of nude mice, with or without addition of IONPs. In the second model, 2 × 10^6^ SK‐Hep‐1 cells suspended in 100 µL 0.9% saline were subcutaneously injected into the right hind limbs of nude mice. The diameters of tumors were measured using calipers once every 3 days. When the tumor volume reached ≈50 mm^3^, the tumor‐bearing mice were tail vein injected with indicated formulation once every 2 to 4 days, for over 11 times. Dosing used: Fe_2_O_3_@DMSA, 40 µg; Fe_2_O_3_@APTS, 40 µg; CQ, 10 mg kg^−1^; and 3‐MA, 80 µmol kg^−1^. The mice were sacrificed 4 days after the final injection, and tissues (heart, liver, spleen, lung, kidney, and subcutaneous tumor) along with blood samples were collected for further analysis. The tumor volumes were calculated using the formula: *V* (mm^3^) = 4/3 × *π* × (shortest diameter)^2^ × (longest diameter)/8.

##### Immuno‐Histochemistry Analysis

Tissues were fully fixed with 10% neutral buffered formalin and embedded in paraffin. The paraffin‐embedded sections were deparaffinized with xylene and rehydrated through graded alcohol series. For histopathological analysis, the paraffin‐embedded sections were stained with H&E.

##### Statistical Analysis

Statistical analysis was compiled on the means of the data obtained from at least three independent experiments with three replicates in each case. All data were expressed as the means ± standard error. The significance of differences in the data of different groups was appropriately determined by the unpaired Student's *t*‐test at *p* < 0.05.

## Conflict of Interest

The authors declare no conflict of interest.

## Supporting information

Supporting Information
