# Supplementary material for: Iron Oxide Nanoparticles as Autophagy Intervention Agents Suppress Hepatoma Growth by Enhancing Tumoricidal Autophagy
Source: Adv Sci (Weinh). 2020 Jun 9;7(16):1903323. doi: 10.1002/advs.201903323 (PMC7435245; doi:10.1002/advs.201903323)
Supplement: Supplementary file 1 — Supporting Information [file ADVS-7-1903323-s001.pdf]

Copyright WILEY-VCH Verlag GmbH & Co. KGaA, 69469 Weinheim, Germany, 2020.

Supporting Information

Iron Oxide Nanoparticles as Autophagy Intervention Agents Suppress Hepatoma Growth by Enhancing Tumoricidal Autophagy

*Yuexia Xie, Jiana Jiang, Qianyun Tang, Hanbing Zou, Xue Zhao, Hongmei Liu, Ding Ma, Chenlei Cai, Yan Zhou, Xiaojing Chen, Jun Pu, and Peifeng Liu**

Table 1

Sequences of q-PCR primers used in this study

Gene	Primer	Sequence
TFRC	Forward	5'-ACCATTGTCATATACCCGGTTCA-3’
	Reverse	5'-CAATAGCCCAAGTAGCCAATCAT-3’
FTH1	Forward	5'-TCCTACGTTTACCTGTCCATGT-3’
	Reverse	5'-GTTTGTGCAGTTCCAGTAGTGA-3’
FTL	Forward	5'-CAGCCTGGTCAATTTGTACCT-3’
	Reverse	5'-GCCAATTCGCGGAAGAAGTG-3’
FPN (SLC40A1)	Forward	5'-CCACAATACGAAGGATTGACCA-3’
	Reverse	5'-ACTGGGGAGCCAAATGTCATA-3’
LC3B	Forward	5'-AAGGCGCTTACAGCTCAATG-3'
	Reverse	5'-CTGGGAGGCATAGACCATGT-3'
P62	Forward	5'-GACTACGACTTGTGTAGCGTC-3'
	Reverse	5'-AGTGTCCGTGTTTCACCTTCC-3'
Beclin1	Forward	5'-GGTGTCTCTCGCAGATTCATC-3'
	Reverse	5'-TCAGTCTTCGGCTGAGGTTCT-3'
Atg2A	Forward	5'-ACACGGAGATCCTGACCTTTC-3'
	Reverse	5'-GGTCGGCTCTTAGGCACAC-3'
Atg16L1	Forward	5'-AACGCTGTGCAGTTCAGTCC-3'
	Reverse	5'-AGCTGCTAAGAGGTAAGATCCA-3'
Atg17	Forward	5'-GAAAGAGCTTGCTCAGGGATT-3'
	Reverse	5'-TCATCAACTGATTTGCGTGACT-3'
Atg18	Forward	5'-AACAGGTCTATGTGCTCTCTCT-3'
	Reverse	5'-CTCATGGGCAGCAATAGTGC-3'
STX17	Forward	5'-TCCTTTGACCAGATCCATGACT-3’
	Reverse	5'-CTTGAGGAATTTCAGGTAAGGCA-3’
SNAP29	Forward	5'-ACTGATGCTTACCCAAAGAACC-3’
	Reverse	5'-GTCCTTCAGACGACCCAGTC-3’
VAMP8	Forward	5'-TGTGCGGAACCTGCAAAGT-3’
	Reverse	5'-CTTCTGCGATGTCGTCTTGAA-3’
GABARAP	Forward	5'-AGAAGAGCATCCGTTCGAGAA-3’
	Reverse	5'-CCAGGTCTCCTATCCGAGCTT-3’
GAPDH	Forward	5'-ACAACTTTGGTATCGTGGAAGG-3’
	Reverse	5'-GCCATCACGCCACAGTTTC-3’

Figure S1

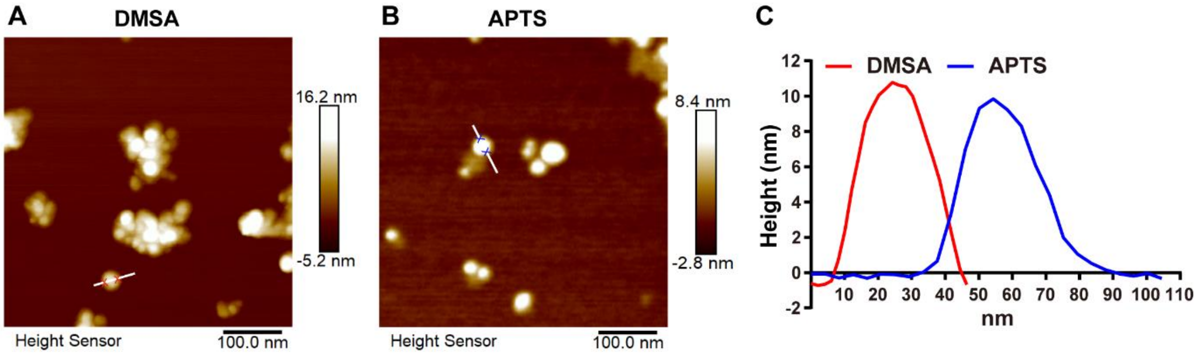

Figure S1. Analysis of Fe_2O_3@DMSA and Fe_2O_3@APTS under atomic force microscopy (AFM). (A and B) AFM images of Fe_2O_3@DMSA and Fe_2O_3@APTS. (C) Typical AFM force curves of single Fe_2O_3@DMSA and Fe_2O_3@APTS particle. DMSA denotes Fe_2O_3@DMSA. APTS denotes Fe_2O_3@APTS.

Figure S2

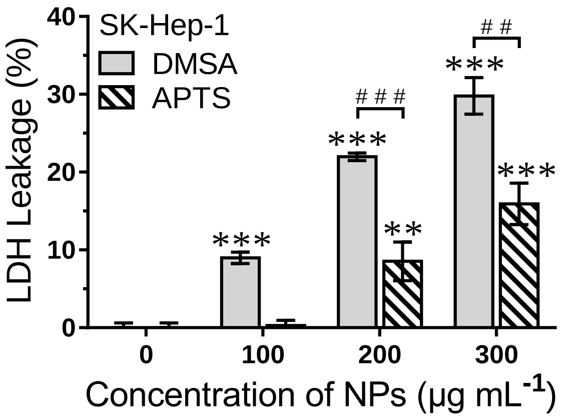

Figure S2. LDH release of SK-Hep-1 cells exposed to gradient concentrations of Fe_2O_3@DMSA or Fe_2O_3@APTS. The data represented mean ± SD. $^{**}p < 0.01$, $^{***}p < 0.001$ compared with control. $^{\#\#}p < 0.01$, $^{\#\#\#}p < 0.001$ between the indicated groups. DMSA denotes Fe_2O_3@DMSA. APTS denotes Fe_2O_3@APTS.

Figure S3

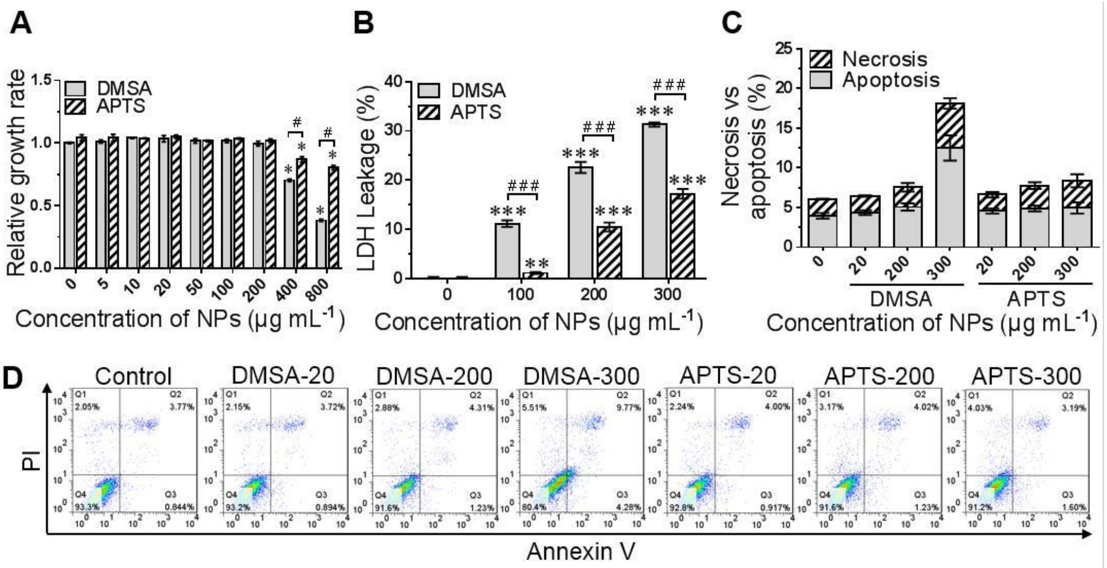

Figure S3. Effect of Fe_2O_3@DMSA and Fe_2O_3@APTS NPs on cytotoxicity in HepG2 cells. (A) Relative cell growth rate of HepG2 cells exposed to gradient concentrations of Fe_2O_3@DMSA or Fe_2O_3@APTS. The data represented mean ± SD. *$p < 0.001$ compared with control. $^{\#}p < 0.001$ between the indicated groups. (B) LDH release of HepG2 cells exposed to gradient concentrations of Fe_2O_3@DMSA or Fe_2O_3@APTS. The data represented mean ± SD. **$p < 0.01$, ***$p < 0.001$ compared with control. $^{\#\#\#}p < 0.001$ between the indicated groups. (C and D) Apoptosis and necrosis of HepG2 cells exposed to 20, 200, or 300 μg mL^{-1} Fe_2O_3@DMSA or Fe_2O_3@APTS. The data represented mean ± SD. DMSA denotes Fe_2O_3@DMSA. APTS denotes Fe_2O_3@APTS.

Figure S4

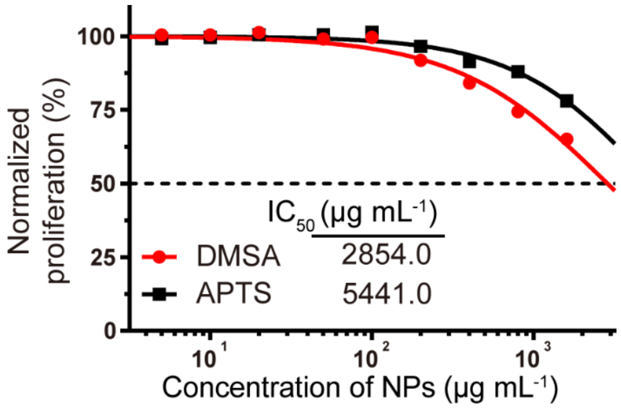

Figure S4. The proliferation rate of normal hepatocytes HL-7702 treated with Fe_2O_3@DMSA and Fe_2O_3@APTS at different concentrations for 24 hours. DMSA denotes Fe_2O_3@DMSA. APTS denotes Fe_2O_3@APTS.

Figure S5

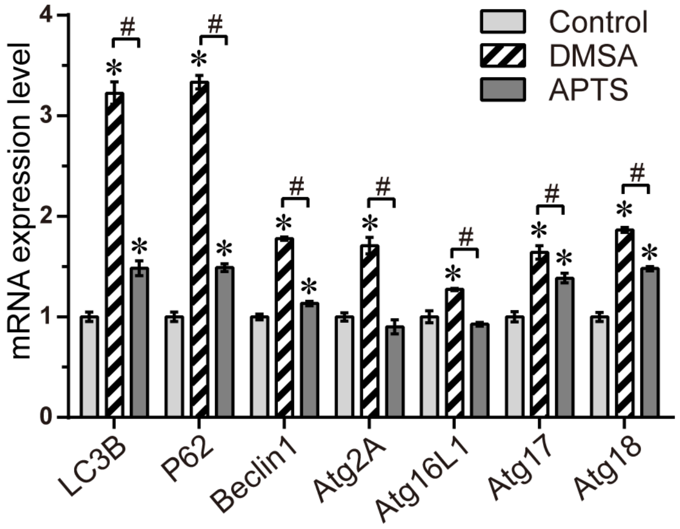

Figure S5. The mRNA expression levels of autophagy markers, including LC3B, P62, Beclin1, Atg2A, Atg16L1, Atg17 and Atg18, were detected by q-PCR in SK-Hep-1 cells exposed to 300 μg mL^{-1} Fe_2O_3@DMSA or Fe_2O_3@APTS. The data represented mean ± SD. *$p < 0.001$ compared with control. $^{\#}p < 0.001$ between the indicated groups. DMSA denotes Fe_2O_3@DMSA. APTS denotes Fe_2O_3@APTS.

Figure S6

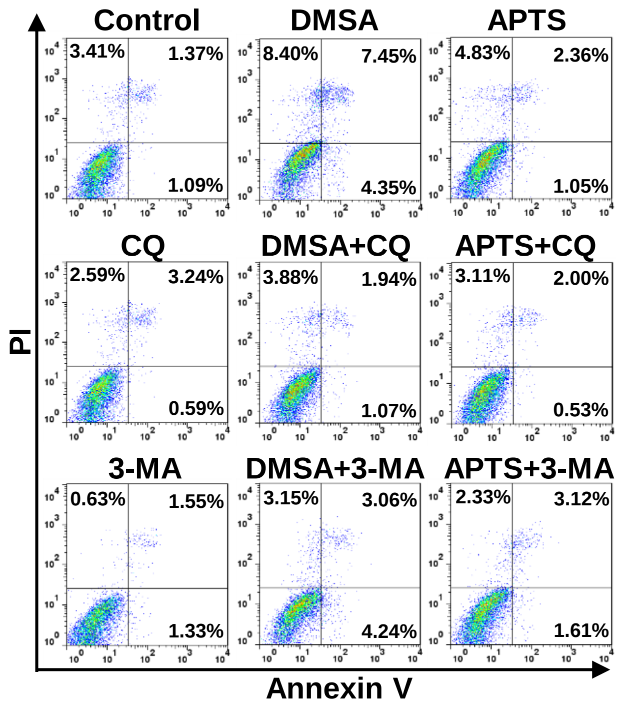

Figure S6. Apoptosis and necrosis of SK-Hep-1 cells exposed to 300 μg mL^{-1} Fe_2O_3@DMSA or Fe_2O_3@APTS with or without CQ or 3-MA. Annexin V and PI staining, and FACS

analysis were performed after treatment for 24 hours. The percentage of annexin V^+ cells represents the apoptotic cells. The percentage of annexin V^+/PI^+ cells represents the necrosis cells. DMSA denotes Fe_2O_3@DMSA. APTS denotes Fe_2O_3@APTS.

Figure S7

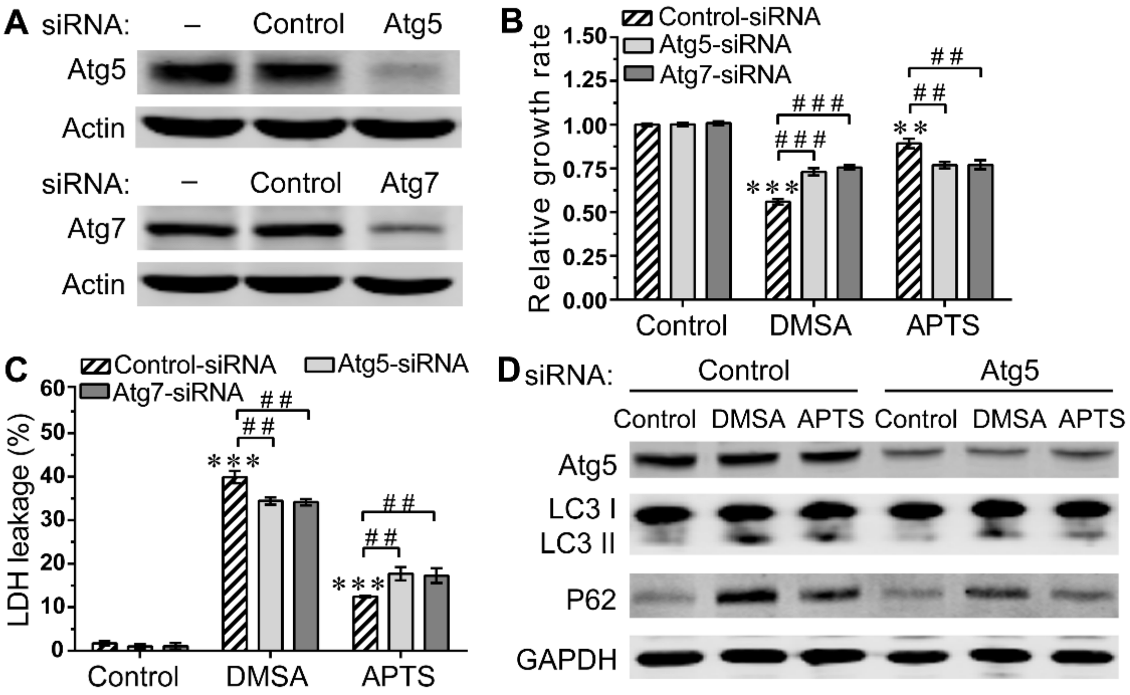

Figure S7. Autophagy exerted reverse effect in Fe_2O_3@DMSA and Fe_2O_3@APTS treated SK-Hep-1 cells. (A) The knockdown efficiency of Atg5 and Atg7 in SK-Hep-1 cells was determined by western blot analysis. (B and C) SK-Hep-1 cells with Atg5/Atg7-knockdown were treated with 300 μg mL^{-1} Fe_2O_3@DMSA or Fe_2O_3@APTS for 24 hour. Relative cell growth rate (B) and LDH release (C) were measured in the groups as indicated. The data represented mean ± SD. $^{**}p < 0.01$, $^{***}p < 0.001$ compared with control. $^{\#\#}p < 0.01$, $^{\#\#\#}p < 0.001$ between the indicated groups. (D) The expressions of LC3 and P62 protein in Atg5 depletion cells exposed to 300 μg mL^{-1} Fe_2O_3@DMSA or Fe_2O_3@APTS. DMSA denotes Fe_2O_3@DMSA. APTS denotes Fe_2O_3@APTS.

Figure S8

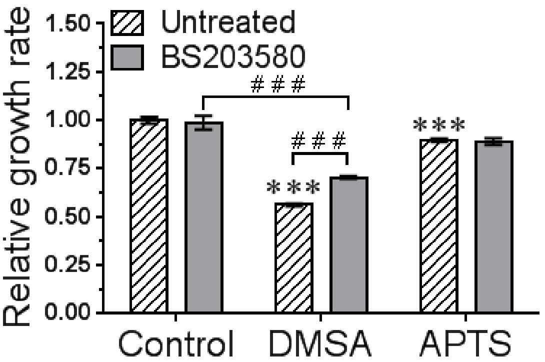

Figure S8. Relative cell growth rate of SK-Hep-1 cells exposed to 300 μg mL^{-1} Fe_2O_3@DMSA or Fe_2O_3@APTS with or without SB203580. The data represented mean ± SD. ***$p < 0.001$ compared with control. $^{\#\#\#}p < 0.001$ between the indicated groups. DMSA denotes Fe_2O_3@DMSA. APTS denotes Fe_2O_3@APTS.

Figure S9

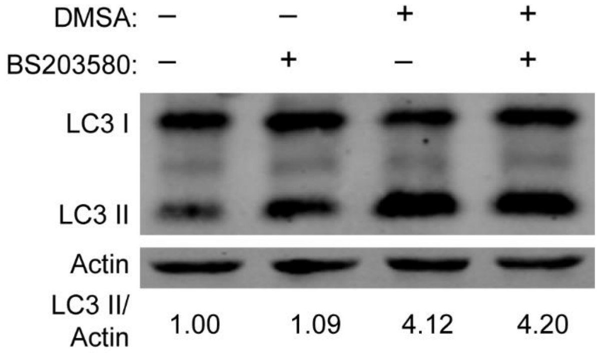

Figure S9. The expressions of LC3 protein in SK-Hep-1 cells exposed to 300 μg mL^{-1} Fe_2O_3@DMSA with or without P38 inhibitor BS203580. DMSA denotes Fe_2O_3@DMSA.

Figure S10

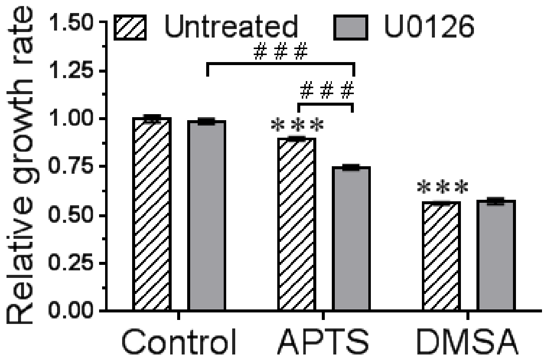

Figure S10. Relative cell growth rate of SK-Hep-1 cells exposed to 300 μg mL^{-1} Fe_2O_3@DMSA or Fe_2O_3@APTS with or without U0126. The data represented mean ± SD. ***$p < 0.001$ compared with control. $^{\#\#\#}p < 0.001$ between the indicated groups. DMSA denotes Fe_2O_3@DMSA. APTS denotes Fe_2O_3@APTS.

Figure S11

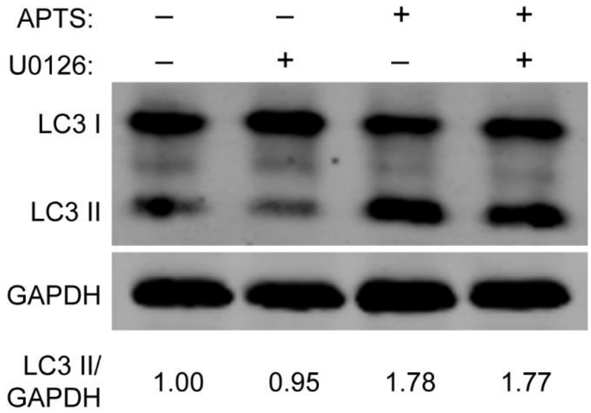

Figure S11. The expressions of LC3 protein in SK-Hep-1 cells exposed to 300 μg mL^{-1} Fe_2O_3@APTS with or without ERK inhibitor U0126. APTS denotes Fe_2O_3@APTS.

Figure S12

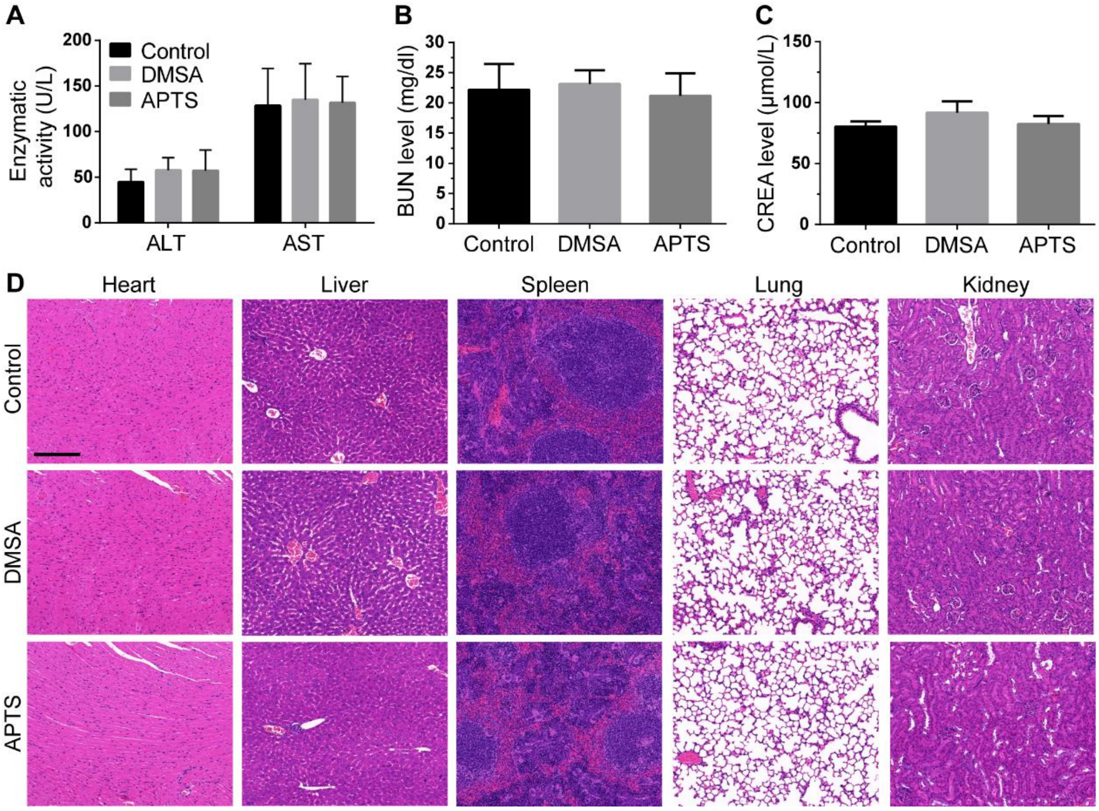

Figure S12. *In vivo* toxicity analysis of SK-Hep-1 xenograft bearing mice treated with Fe_2O_3@DMSA and Fe_2O_3@APTS, respectively. (A to C) Serum levels of ALT, AST, BUN and CREA of mice were detected. (D) Hematoxylin and eosin (HE) staining of organs, including heart, liver, spleen, lung and kidney from mice. The scale bar is 200 μm. DMSA denotes Fe_2O_3@DMSA. APTS denotes Fe_2O_3@APTS.
